# Dietary cellulose induces anti-inflammatory immunity and transcriptional programs via maturation of the intestinal microbiota

**DOI:** 10.1080/19490976.2020.1829962

**Published:** 2020-10-20

**Authors:** Florence Fischer, Rossana Romero, Anne Hellhund, Uwe Linne, Wilhelm Bertrams, Olaf Pinkenburg, Hosam Shams Eldin, Kai Binder, Ralf Jacob, Alesia Walker, Bärbel Stecher, Marijana Basic, Maik Luu, Rouzbeh Mahdavi, Anna Heintz-Buschart, Alexander Visekruna, Ulrich Steinhoff

**Affiliations:** a Institute for Medical Microbiology and Hospital Hygiene, Philipps University, Marburg, Germany; b Core Facility for Mass Spectrometry and Elemental Analysis, Philipps University, Marburg, Germany; c Institute for Lung Research, Universities of Giessen and Marburg Lung Center, Philipps University, Marburg, Germany; d Institute of Anatomy and Cell Biology, Philipps University, Marburg, Germany; e Experimental Animal Facility, Biomedical Research Center, Philipps University, Marburg, Germany; f Department of Cell Biology and Cell Pathology, Philipps University, Marburg, Germany; g Research Unit Analytical BioGeoChemistry, Helmholtz Zentrum München, Munich, Germany; h Max Von Pettenkofer-Institute for Hygiene and Clinical Microbiology, Ludwig Maximilians-University München and German Center for Infection Research (DZIF), Partner Site Munich, Munich, Germany; i Institute of Laboratory Animal Science, Hannover Medical School, Hannover, Germany; j Department Soil Ecology, Helmholtz Centre for Environmental Research - UFZ, Halle/Saale, Germany

**Keywords:** Cellulose, insoluble fiber, microbiota maturation, microbial diversity, bile acids, mucosal homeostasis, inflammation, Alistipes, Reg3γ, IL-22

## Abstract

Although it is generally accepted that dietary fiber is health promoting, the underlying immunological and molecular mechanisms are not well defined, especially with respect to cellulose, the most ubiquitous dietary fiber. Here, the impact of dietary cellulose on intestinal microbiota, immune responses and gene expression in health and disease was examined. Lack of dietary cellulose disrupted the age-related diversification of the intestinal microbiota, which subsequently remained in an immature state. Interestingly, one of the most affected microbial genera was *Alistipes* which is equipped with enzymes to degrade cellulose. Absence of cellulose changed the microbial metabolome, skewed intestinal immune responses toward inflammation, altered the gene expression of intestinal epithelial cells and mice showed increased sensitivity to colitis induction. In contrast, mice with a defined microbiota including *A. finegoldii* showed enhanced colonic expression of intestinal IL-22 and Reg3γ restoring intestinal barrier function. This study supports the epidemiological observations and adds a causal explanation for the health promoting effects of the most common biopolymer on earth.

## Introduction

Over recent decades, dietary habits in industrialized countries have changed substantially. The westernized diet is characterized by a high content of protein and fat but low dietary fiber as opposed to diets in traditional societies.^1^ The paradigmatic shift that diets low in fiber are often associated with an increased incidence of “lifestyle diseases”, such as coronary heart disease, diabetes and certain gastrointestinal disorders, was first proposed by Burkitt and colleagues.^2,3^ Today, numerous epidemiological, clinical and experimental studies have confirmed the health promoting effects of dietary fiber.^4^

Dietary fibers include a highly heterogenous group of carbohydrate polymers with different physicochemical properties that are indigestible by enzymes produced by the gastrointestinal tract.^5^ As dietary fibers are neither digested nor absorbed, they reach lower regions of the intestine and serve as substrate for the metabolically active gut microbes. While short-chain fatty acids (SCFAs), a bacterial fermentation product of many fibers, have been shown to beneficially influence the mucosal immune system and barrier function, the biological effects of the non-fermentable fiber, such as cellulose, are not yet understood.

Dietary cellulose is an insoluble fiber and consists exclusively of unbranched β-1,4-linked glucose monomers. It is the major component of plant cell walls and thus a prominent fiber in grains, vegetables and fruits. Whereas the importance of cellulolytic bacteria for ruminants was described already in the 1960s, it still remains enigmatic whether the fermentation of cellulose has physiological effects in monogastric mammals.^6–11^ Under experimental conditions, it has been shown that the amount of dietary cellulose influences the richness of the colonic microbiota, the intestinal architecture, metabolic functions and susceptibility to colitis.^12,13^ Moreover, mice fed a cellulose-enriched diet were protected from experimental autoimmune encephalomyelitis (EAE) through changes in their microbial and metabolic profiles and reduced numbers of pro-inflammatory T cells.^14^

The aim of the current study was to selectively investigate the effects of dietary cellulose with regard to microbial, molecular and immunological responses in the absence of any other fiber. Thus, experiments were performed with two chemically defined diets, one of which was devoid of any fiber while the other contained cellulose as the only source of nutritional fiber.

The results show that cellulose is fermented by members of the colonic microbiota and that lack of this fiber impedes diversity development of the microbiota, the genus *Alistipes* being most affected. Further, feeding cellulose impacted gene expression by colonic epithelial cells and intestinal barrier function. Using gnotobiotic mice harboring a defined microbiota, it was demonstrated that *A. finegoldii* mediated protection from colitis mainly through induction of IL-22 and Reg3γ in the colon. In summary, the data provide a mechanistic link between specific members of the gut microbiota and profound effects of dietary cellulose on human health.

## Results

### Maturation of the intestinal microbiota during early development requires cellulose

To specifically study the effects of dietary cellulose on the microbiota, mice were fed from birth with purified diets which were identical except that one was completely fiber-free (FFD) and the other contained 7% cellulose as the only source of dietary fiber (CD). The effect of dietary cellulose deficiency on body weight and intestinal transit time was examined. As expected, intestinal transit time was increased in CD animals but the weight gain of mice was similar in CD and FFD groups, showing that cellulose did not affect the growth of mice (Figure S1).

**Figure 1.**
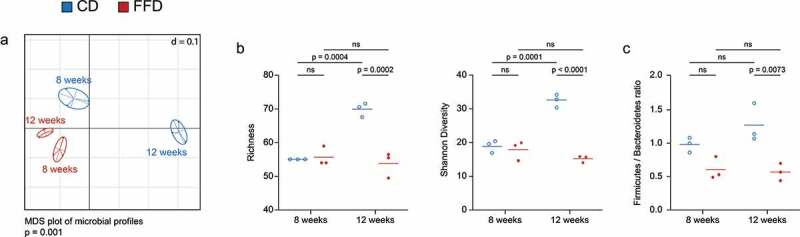
The impact of cellulose on the diversification of the intestinal microbiota. The diversity of the intestinal microbiota of eight and twelve-week-old B6 mice was analyzed by 16S rRNA gene amplicon analysis. (a) Diversity shown as multi-dimensional scaling (MDS) plot based on generalized UniFrac dissimilarities (n = 3). (b) Alpha diversity shown as richness and Shannon diversity and (c) the Firmicutes/Bacteroidetes ratio (n = 3). Statistical analysis: (a) non-parametric multivariate analysis of variance (Rhea), (b, c) one-way ANOVA. Data are shown as individual mice and means and are representative of two independent experiments

Although cellulose is not a classical prebiotic, the possibility that it exerts these effects by impacting the microbiota was examined via 16S rRNA gene amplicon analysis in mice at the age of 8, 12 and 25 weeks. Beta diversity analysis illustrated that diet and age significantly influence diversification of the microbiota between week 8 and 12, as both factors led to distinct clustering (Figure 1a). In contrast to FFD, an increase of microbial diversification and Firmicutes/Bacteroidetes ratio as marker for microbiota maturation^15^ in CD occurred between weeks 8 and 12. Interestingly, between weeks 12 and 25, differences in alpha and beta diversity were less pronounced but still significant (Figure S2). This indicates that microbial diversification was largely completed in 12-week-old CD animals, but remained immature in FFD mice.

**Figure 2.**
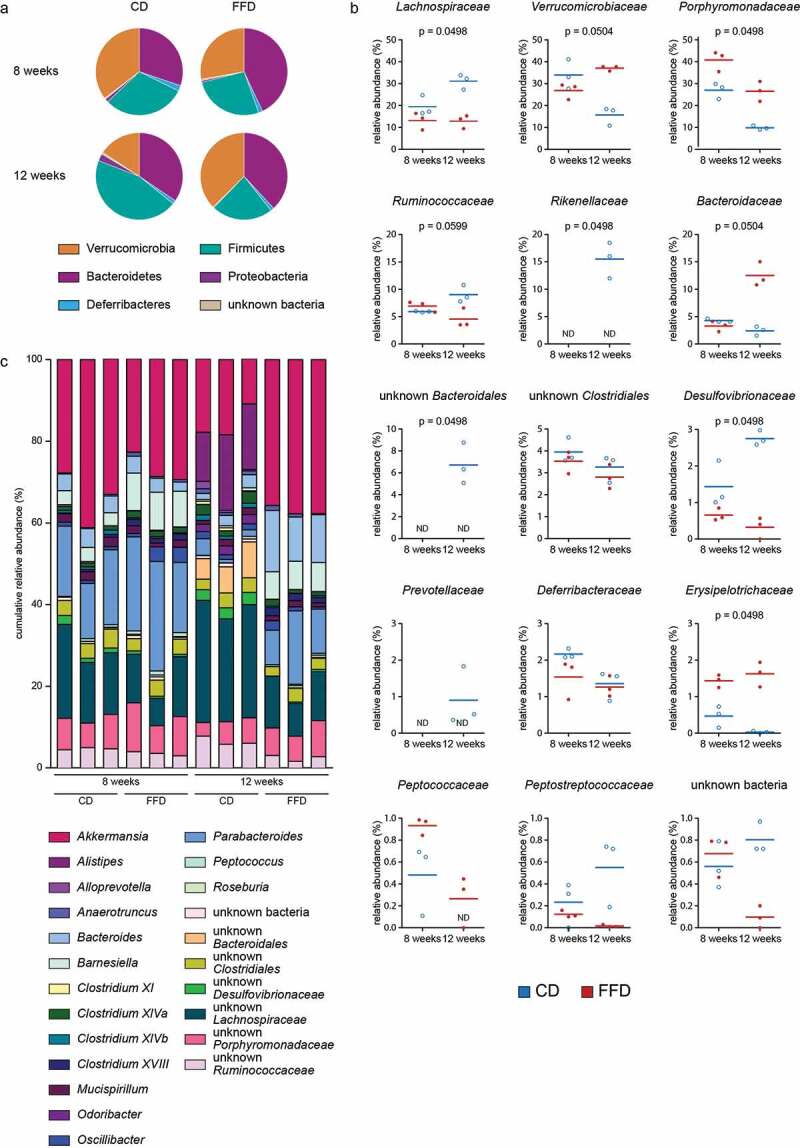
Alterations of intestinal microbiota at taxonomic levels. Taxonomic composition of the intestinal microbiota of CD or FFD mice. (a) Phyla, (b) family and (c) genera at the age of eight and twelve weeks was measured via 16S rRNA gene amplicon analysis (n = 3). Statistical analysis: (b) Kruskal-Wallis Rank Sum Test for all groups (corrected for multiple comparison by Benjamini-Hochberg method). Data are shown as individual mice and means and are representative of two independent experiments

To identify bacterial taxa most sensitive to cellulose deprivation, the relative abundance of the colonic microbiota at various taxonomic levels at weeks 8 and 12 was examined. The impact of cellulose was evident at the level of phyla but also at lower taxonomic levels, i.e. families and genera (Figure 2). FFD mice showed an increased relative abundance of the families *Verrucomicrobiacaea, Porphyromonadaceae* and *Bacteroidacea* and a decrease in *Lachnospiraceae, Ruminococcaceae* and *Desulfovibrionaceae*. Most importantly, *Rikenellaceae*, a dominant family of CD mice, was below the detection limit in the FFD group, due to the disappearance of the genus *Alistipes*, represented by *Alistipes finegoldii* 17242.

We further wondered, whether the shift of microbiota in the absence of dietary cellulose is also reflected by the bacterial metabolome. While fermentation of dietary fiber often results in the production of SCFA, the presence of cellulose did not enhance the concentrations of, for example, acetic, butyric, propionic and valeric acid (Figure S3a) and were not detectable in germfree mice (GF) independently of CD or FFD. By contrast, the pool of cecal bile acids was highly affected by cellulose. The amounts of primary bile acids were elevated in germ-free mice kept on FFD as compared to those kept on CD (Figure S3b) and did not differ in SPF animals, indicating a bacteria-independent mechanism. However, the primary unconjugated bile acid UDCA and the secondary bile acids DCA and 3-dehydroCDCA were significantly increased in SPF FFD mice (Figure S3c, d).

In summary, these data show that cellulose drives microbial diversity and maturation during early adulthood of mice and impacts on the gut bile acid metabolism.

### Cellotetraose is cleaved by intestinal microbial enzymes

**Figure 3.**
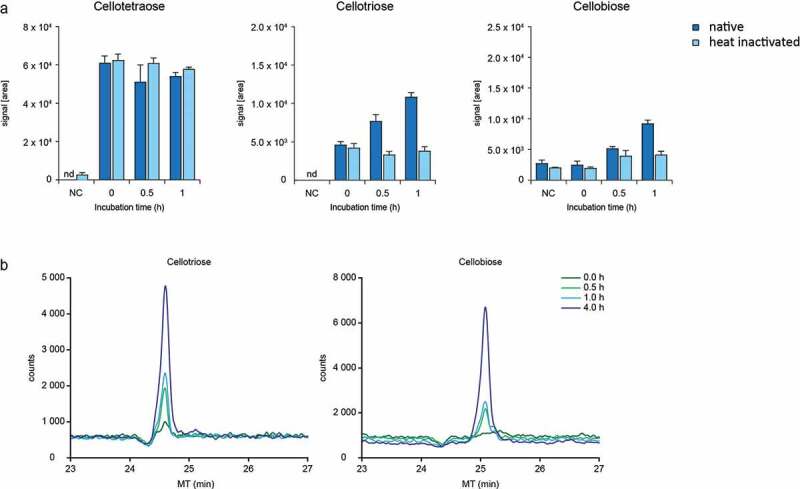
Degradation of cellotetraose by enzymes of the cecal microbiota. (a) Signals intensity (AUC) measured by HPLC-MS and (b) CE-MS electropherogram of degradation products after incubation of cellotetraose with native and heat-inactivated cecal enzymes of CD mice (n = 1). Data are shown as means ± SD and are representative of two independent experiments

The strong impact of cellulose on the microbiota of the colon suggests that this fiber might serve as substrate for bacterial metabolism. Although humans and animals lack endogenous enzymes necessary to break down the β-1,4-glycosidic bonds of cellulose, some bacteria have been shown to exert cellulolytic activity.^8^ Therefore, the possibility that the intestinal microbiota of both CD and FFD animals generate enzymes able to cleave β-1,4-glycosidic bonds was examined. Supernatants derived from cecal contents of both dietary groups were incubated with water soluble cellotetraose which is suitable for enzymatic assays. Analysis of the degradation products by CE- and HPLC-MS showed that native, but not heat inactivated, supernatants degraded cellotetraose into cellotriose and cellobiose (Figure 3a,b).

In-vitro cleavage of cellotetraose was also seen in FFD animals indicating that their less diverse microbiota was able to cleave cellulose (Figure S4a). As expected, cleavage of cellotetraose was strictly microbe dependent, as cecal supernatants from germfree mice were not able to degrade cellotetraose (data not shown). These findings implicate that cellulose is a substrate that fuels microbial development.

### Dietary cellulose modulates the function of intestinal immune and epithelial cells

**Figure 4.**
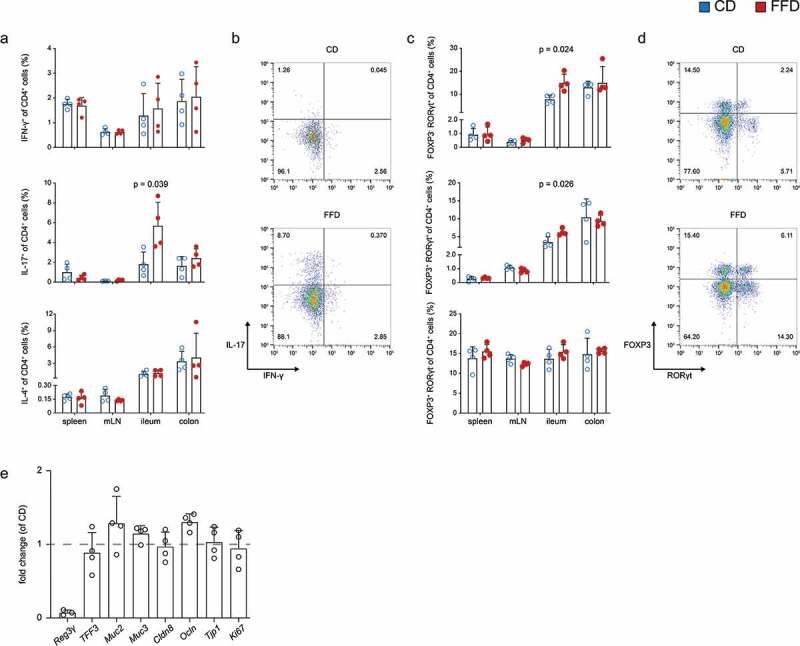
Influence of dietary cellulose on the intestinal immune and epithelial cells. T-lymphocytes isolated from spleen, mLN and intestinal lamina propria of CD and FFD mice were analyzed by FACS. (a) Cytokine secretion in indicated organs and (b) representative plots for ileal CD4^+^ T cells (n = 4). (c) Expression of transcription factors in indicated organs and (d) representative plots for transcription factor expression in ileal CD4^+^ T cells (n = 4). (e) RT-PCR of indicated genes from colonic tissue of FFD mice normalized to CD animals (n = 4). Statistical analysis: (a, c) Welch’s test. Data are shown as individual mice and means ± SD and are representative of two independent experiments

As microbes and diet are known to impact intestinal immune responses, the influence of cellulose on the balance of pro- and anti-inflammatory lymphocytes was examined in various tissues.^16,17^ While absolute lymphocyte numbers were unaffected, ileal tissues of FFD mice contained increased frequencies of CD4^+^ T cells (Figure S5a,b). Cytokine expression revealed enhanced frequencies of ileal IL-17^+^ but not IFN-γ or IL-4 secreting CD4^+^ T cells in the absence of cellulose (Figure 4a,b). This was confirmed by staining of the transcription factor RORγt, known to induce pro-inflammatory T_H_17 differentiation (Figure 4c,d). Interestingly, intestinal frequencies of FOXP3^+^ RORγt^−^ CD4^+^ Tregs and the amount of sIgA involved in mucosal homeostasis were not affected by dietary cellulose (Figure S5c).

Further, the possibility that cellulose influences the expression of selected genes involved in intestinal barrier function and homeostasis was measured. RT-PCR of colonic tissues revealed that lack of cellulose in FFD mice particularly reduced the expression of the antimicrobial protein, regenerating islet-derived protein 3 gamma (Reg3γ) (Figure 4e). Mucins, tight junction proteins and the proliferation marker Ki76 were not or only marginally affected under homeostatic conditions.

### Dietary cellulose protects from colitis

**Figure 5.**
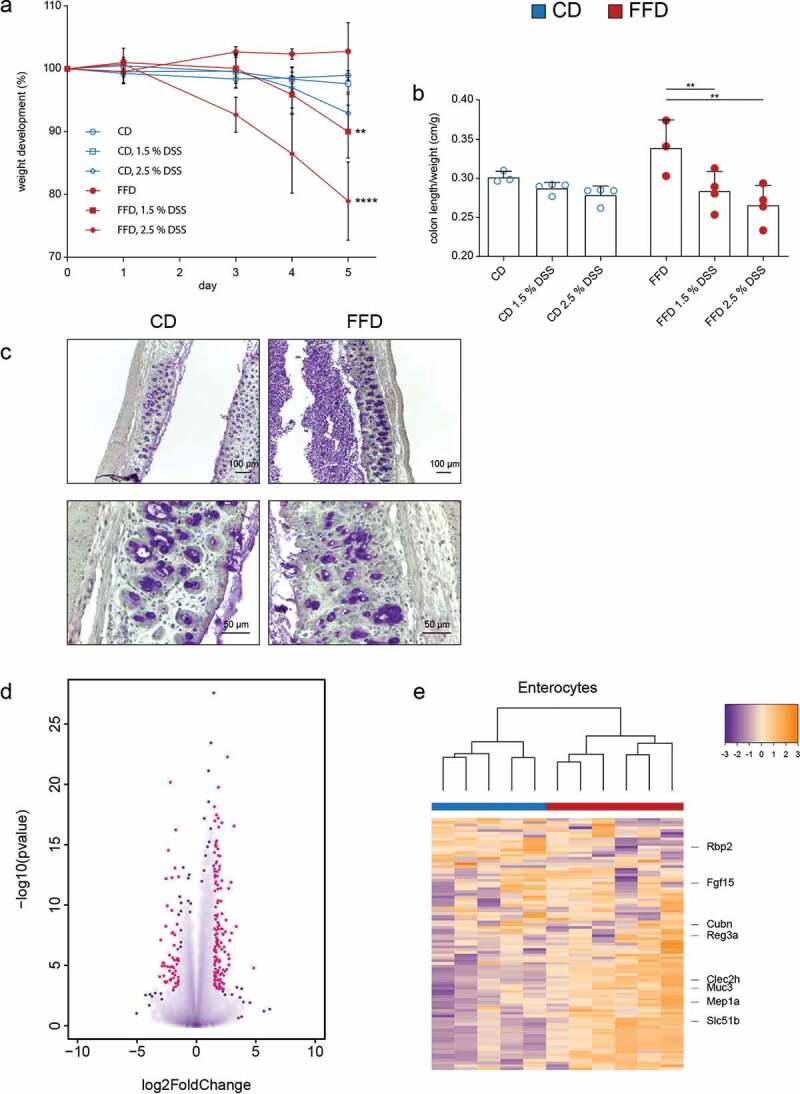
Influence of dietary cellulose on development of colitis and transcriptional profiles of gut epithelial cells. (a) Weight loss and (b) colon length/weight ratio in CD or FFD mice after treatment with DSS (1.5% and 2.5%) in the drinking water. (c) Periodic acid-Schiff histology (PAS) of the colon (2.5% DSS) (n – 3-4). (d) Differentially expressed genes of colonic epithelial cells of CD or FFD RAG KO mice after treatment with DSS (1.5%) for five days; red points represent genes statistically differentially expressed with *p* = .005 and log fold change > 1.5 (FFD vs CD). (e) Unsupervised clustering of genes related to distinct intestinal enterocytes differentially expressed in CD and FFD RAG KO mice (n = 5–6). Statistical analysis: (a) two-way ANOVA (* are related to weight loss at day five in comparison to untreated mice), (b, d) multiple t test corrected by Benjamini-Hochberg-method. *p < .03, **p < .002, ***p < .0002, and ****p < .0001. Data are shown as individual mice and means ± SD and are representative of two independent experiments

Dysbiosis and metabolic changes of the intestinal microbiota often results in inflammation.^18,19^ Possible functional consequences for colitis development were therefore examined. Comparing CD and FFD animals in an experimental colitis model, FFD mice revealed increased susceptibility for intestinal inflammation even at low concentrations of DSS (1.5 %) as evaluated by weight loss, diarrhea and reduced colon length (Figure 5a, b; Figure S6a,b). DSS caused loss of crypt structure, epithelial damage and massive infiltration of immune cells (Figure 5c). In accordance, increased amounts of colonic TNF-α and lipocalin-2 in FFD mice further demonstrated the protective effect of dietary cellulose on DSS-induced colitis (Figure S6c).

As inflammation activates complex transcriptional programs, we wondered whether dietary cellulose may affect gene expression in the gut epithelium even under such harsh conditions.^20^ Thus, RNA-Seq analysis of colonic epithelial cells isolated from CD and FFD mice after DSS treatment was performed. To ensure that gene signatures were derived from epithelial cells and not from contaminating lymphocytes, mice deficient in intraepithelial lymphocytes (RAG KO) were used. Differential gene expression analysis revealed that cellulose had multiple effects on the transcriptional profile of colonic epithelial cells (Figure 5d). Furthermore, the differential gene expression pattern was assigned to distinct epithelial cell types, according to recently published cell-specific signatures.^21^ Cellulose deficiency caused a distinct clustering, particularly pronounced in enterocytes, tuft-, goblet- and enteroendocrine cells (Figure 5e; Figure S6d). Of note, despite the lack of adaptive immune cells, cellulose fed RAG KO mice showed increased resistance against colitis as compared to cellulose-free animals, a situation comparable to immunocompetent mice (Figure S6e, f). These data demonstrate that dietary cellulose triggers transcriptional programs during homeostasis and inflammation.

### A. finegoldii mimics the anti-inflammatory effect of cellulose

**Figure 6.**
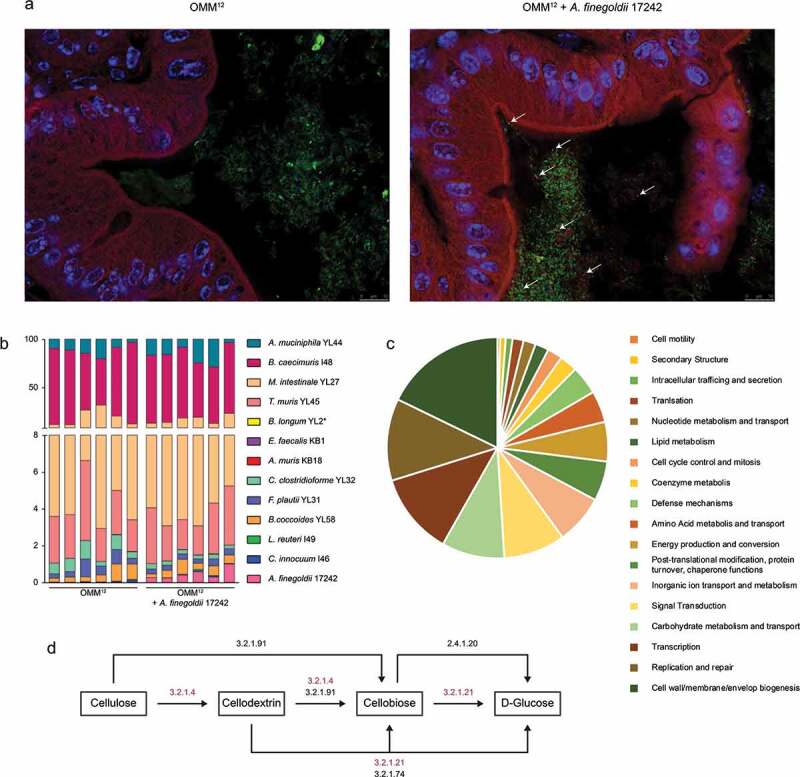
*A. finegoldii* enriches the Oligo-MM^12^ with cellulolytic potential. Oligo-MM^12^ mice were colonized with *A. finegoldii* 17242 and (a) viable *A. finegoldii* (white arrows) was visualized by FISH in the cecum (green, all bacteria; red, *A. finegoldii*; blue, DAPI). (b) Relative abundance of *A. finegoldii* within the Oligo-MM^12^ consortium was quantified by RT-PCR (n = 6). (c) Singletons were assigned to cluster of orthologous genes (EDGAR). (d) Scheme of the cellulose degrading pathways. Genes encoding cellulose degrading enzymes marked in red were exclusively found in *A. finegoldii* but not Oligo-MM^12^

Because fiber deficiency led to a strong reduction of *A. finegoldii*, the causal relationship of this bacterium to cellulose-mediated protection via the microbiome was examined. For functional studies on host-microbe interaction, Oligo-MM^12^ mice which allow tracing of individual bacteria in the presence of a mature immune system were used. These mice harbor a defined community of 12 bacterial strains which represent the most prevalent bacterial phyla in the murine gut.^22^ Association of Oligo-MM^12^ mice with *A. finegoldii* resulted in stable colonization of the cecum and colon without disrupting the original Oligo-MM^12^ microbiota, as shown by FISH and quantitative PCR analysis (Figure 6a,b). Further, comparative genome analysis with EDGAR^23^ showed that *A. finegoldii* enriched the Oligo-MM^12^ metagenome with 1,653 genes (singletons), related to cell wall and membrane metabolism (M), genetic information processing (K, L) and carbohydrate metabolisms (G) (Figure 6c). In addition, KEGG pathway analysis revealed that *A. finegoldii* is endowed with unique genes involved in cellulose degradation, possibly explaining its preponderance in a cellulose containing habitat (Figure 6d).

**Figure 7.**
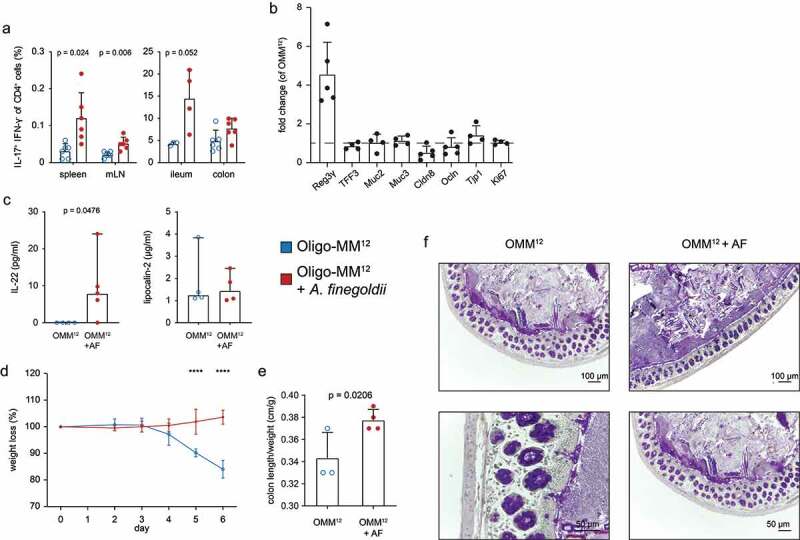
*A. finegoldii* enhances the intestinal barrier and protects from colitis. Oligo-MM^12^ mice were colonized with *A. finegoldii* three weeks prior to analysis. (a) IL-17 and IFN-γ secretion in CD4^+^ T cells in indicated organs was measured via FACS (n = 4–6). (b) RT-PCR of indicated genes from colon normalized to Oligo-MM^12^ mice without *A. finegoldii*. (c) Cytokines from colon cultures measured by LEGENDplex^TM^ and ELISA. Impact of *A. finegoldii* on (d) weight loss and (e) colon length/weight ratio of Oligo-MM^12^ after DSS treatment (3.5%) (n = 3–4). (f) Periodic acid-Schiff histology (PAS) of the colon from Oligo-MM^12^ and Oligo-MM^12^ + *A. finegoldii* mice (n = 2). Statistical analysis: (a) Welch’s test, (c) Mann-Whitney Test, (d) two-way ANOVA and (e) Student’s t test. *p < .03, **p < .002, ***p < .0002, and ****p < .0001. Data are shown as individual mice and (a, b, d, e) means ± SD or (c) median ± CI and are representative of two independent experiments

Recent studies have shown that the commensal microbiota is able to influence the transcriptional profile of intestinal epithelial and immune cells.^24–26^ The possibility that the presence of *A. finegoldii* within the Oligo-MM^12^ microbiota alters gene expression and favors intestinal homeostasis was therefore examined. Analogous to SPF mice, immunological markers and expression of genes essentially involved in gut barrier function were measured. *A. finegoldii* enhanced the frequency of IL17-producing T cells in the intestine and peripheral lymphoid organs (Figure 7a; Fig. S7a-f) and induced a strong and specific upregulation of Reg3γ in the colon (Figure 7b). Colon cultures further revealed selective upregulation of IL-22 in *A. finegoldii*-associated Oligo-MM^12^ mice. The inflammation marker lipocalin-2 was not affected under these conditions (Figure 7c, Fig. S7g). Together, IL-22 and Reg3γ, are two factors known to maintain the barrier via segregating the microbiota from the intestinal epithelium.^27,28^

Finally, the barrier-enhancing property of *A. finegoldii* was examined in DSS colitis and revealed reduced intestinal inflammation in *A. finegoldii* associated Oligo-MM^12^ mice. This was evidenced by the lack of weight loss, increased colon length, and low amounts of TNF-α and Lipocalin-2 compared to the control group (Figure 7d, e Fig. S7h). Further, PAS staining of colonic tissue revealed only mild signs of colitis with reduced mucus secretion, less cellular infiltrates and thickening of the mucosal tissue (Figure 7f).

## Discussion

The rise in non-communicable diseases, such as diabetes, IBD, asthma and cancer is often linked to the relationship between diet, microbiota and immunity.^17,29^ Several epidemiological and experimental studies have shown that the health benefits of dietary fibers are related to the composition and function of the intestinal microbiota.^30–32^ However, differences in the amount and type of plant derived polysaccharides associated either with short-term or life-long alterations of the microbiota make comparisons difficult.^33–35^ For this reason, the current study specifically investigated the effects of cellulose, the most abundant dietary fiber, by feeding normal and gnotobiotic mice fiber-free diets or diets containing cellulose as only fiber source.

The study revealed that the polymer cellulose is cleaved by intestinal bacteria into oligomers and finally glucose which are used as substrate and promote the development of a mature-like microbiota. Digestion of cellulose requires specific enzymes that have been described for several bacterial genera, including *Xylanibacter, Prevotella, Butyrivibrio, Bacteroidales* and *Faecalibacterium*, most of which are abundantly present in the microbiota of people living in rural Africa. Far more fibers are consumed in agricultural than industrialized societies.^31,36^

Since the early eighties, studies have shown that despite its limited fermentation to SCFA, cellulose is able to substantially modify the colonic microbiota, classifying cellulose as a potential prebiotic.^37,38^ The metabolism of cellulose in man has long been subject of scientific interest. Several studies with ^14^C -labeled cellulose have shown the difficulties of quantifying the metabolism of cellulose in humans. The majority of ^14^C was recovered from feces and breath, but isotopes could be present in various chemical forms which are difficult to determine.^39^ Thus, cellulose degradation in humans remains controversial. The present study shows that dietary cellulose fuels microbial diversity and influences the bacterial metabolome, i.e. secondary bile acids. Although lack of dietary fiber affected several microbial taxa, *Alistipes spp*., a dominant genus of the human core microbiota, were not detectable in the absence of dietary fiber.^33^

Nutrient availability is a fundamental factor that dictates the establishment of microbial communities and their metabolic interactions. Maturation of the microbiota occurs stepwise, first after birth and lactation, followed by the weaning period with increased ingestion of solid foods.^40,41^ Antigen encounter (bacterial and food) during weaning, the so called weaning reaction, and transition from the postnatal to the adult period has shown to be critical for the development of the mucosal immune system.^42–44^ This is in accordance with the current view that the development of the gut microbiota takes more time than previously thought.^45^ The current findings demonstrate that dietary cellulose is essential for the advancement of microbial diversification beyond eight weeks of life, indicating that cellulose promotes the progression from an early, infant-like microbiota toward an adult-type complex microbiota in the absence of other fiber and further decreased sensitivity to develop intestinal inflammation.

As the intestinal immune system is essentially involved in maintenance of gut homeostasis, we also investigated whether dietary cellulose impacts on the adaptive immune system. In contrast to a recent study, no influence on Th1 and Th2 cells was observed in the current study.^14^ However, fiber deficiency was associated with increased frequencies of pro-inflammatory Th17 cells, most likely a consequence of dysbiosis or altered composition of bile acids in FFD animals.^46–48^ In addition, regulatory T cells (T_reg_) known to contribute to intestinal homeostasis were similar in CD and FFD mice, supporting that cellulose contributes to only small amounts of SCFA, known to promote T_reg_ development.^49–51^

While it is accepted that the microbiota is connected with various states of health or disease, it often remains challenging to establish the causality and mechanisms of such host-microbe interaction. Recently, co-culture experiments of human colonic epithelial cells with a live microbiota demonstrated how defined bacterial genes alter the transcriptional response in epithelial cells.^24^ In support of this, the current study shows that dietary cellulose alters the microbiota and consequently the transcriptional program of various epithelial cell types not only during inflammation but also under homeostatic conditions.

The present work highlights that dietary cellulose shows profound effects in a complex, as well as defined model microbiota and suggests that degradation products of cellulose act on microbial composition. Here, *A. finegoldii* has a key position, as this species is most susceptible to cellulose deprivation and provides protection against colitis if transferred into Oligo-MM^12^ mice. In silico analyses revealed that the genome of *A. finegoldii* encodes for enzymes required for the breakdown of cellulose into glucose, supporting previous data that in vitro culture of *A. finegoldii* was enhanced in the presence of cellulose.^52^ Even though *A. finegoldii* has been controversially discussed concerning its beneficial or harmful implications, it should be noted that this organism is present in healthy as well as inflamed tissues.^53–57^ This shows that the interaction of specific microbes with their host is highly contextual, the same microbe may behave as mutualist or pathogen according to the nutrition, composition of the microbiota, coinfection or genetic landscape of its host. Here, the Oligo-MM^12^ mouse model allowed investigation of the influence of *A. finegoldii* in a defined microbial setting. Molecular quantification of bacterial members of the microbiota revealed that *A. finegoldii* stably colonized without disrupting the Oligo-MM^12^ microbiota. Surprisingly, *A. finegoldii* did not entirely mimic the effect of cellulose but also induced Th17 immunity. Recently, two distinct intestinal Th17 subset have been described: Homeostatic IL-17A- and IL-22-secreting Th17 cells induced by commensal segmented filamentous bacteria (SFB) and IL-22- and IFN-γ-producing, inflammatory Th17 cells, triggered by pathogenic *C. rodentium*.^58^ Here, *A. finegoldii* induced a Th17 response similar to SFB, with increased IL-22 but unchanged levels of IFN-γ and lipocalin-2.

Of note, innate lymphoid cells (ILC) namely ILC3 which also express the transcription factor RORγt^59^ are another potential source of IL-22. These cells start to secrete IL-22 after stimulation with enteric commensals.^60^ It is subject of further studies to disclose which IL-22-producing cell type is affected by *A. finegoldii*.

IL-22 signaling to intestinal epithelial cells induces antimicrobial proteins Reg3β and Reg3γ and mucin production, both important for a stable mucus layer and to avoid dysbiosis.^28^ Further, IL-22 supports healing and regeneration of the intestinal epithelium after damage.^61,62^ Of note, the presence of *A. finegoldii* within the minimal microbiota was sufficient to induce Reg3γ and IL-22, factors known to support a strong intestinal barrier.

Taken together, the study demonstrated the potential of dietary cellulose to induce cellular and molecular anti-inflammatory mechanisms via maturation of the intestinal microbiota and thus provides an immunological and molecular rationale for the health benefits of cellulose.

## Material and methods

### Animals

C57BL/6 mice (Charles River Laboratories) and RAG1 KO mice were bred under specific pathogen-free (SPF) conditions at the animal facilities at the University of Marburg. Germ-free C57BL/6 mice (GF) and Oligo-MM^12^ mice were kept in sterile plastic isolators (Metall and Plastik, Germany) with autoclaved food, bedding and water at the animal facilities at the University of Marburg. Sterility of GF was checked biweekly by culturing feces in thioglycollate medium under aerobic and anaerobic conditions for at least 10 days. All handling procedures for GF and Oligo-MM^12^ mice, were conducted in a laminar flow hood under sterile conditions. GF mice generated via rederivation through cesarean section were a kind gift of Dr. P. Kirsch (University of Ulm). Oligo-MM^12^ mice were kindly provided by Dr. M. Basic, central animal facility of the Hannover Medical School (MHH). All experiments were conducted in accordance with German animal protection law.

### Animal nutrition

C57BL/6 mice (SPF) were kept from birth on a control diet with 7% cellulose (CD) (S7242-E014; Sniff) or fiber-free diet (FFD) (S7242-E018; Sniff). RAG KO mice were kept for 4 weeks on CD or FFD diets prior to DSS treatment. Diets contained all essential vitamins, minerals, trace elements, fat, dextrin, sucrose and free amino acids equimolar to the protein content of normal rodent chow (Fig. S1a). Gnotobiotic Oligo-MM^12^ and germfree C57BL/6 mice were kept on LASQCdiet Rod16-R from LASvendi.

### Culture and gavage of bacteria

*Alistipes finegoldii* 17242 was purchased from the German Collection of Microorganisms and Cell Culture GmbH (DSMZ, Braunschweig, Germany) and cultured under anerobic conditions at 37°C for three to four days in BHI media. For association of mice, 200 µl of *A. finegoldii* 17242 was administered orally by gavage, controls received 200 µl native BHI medium. For stable colonization, mice were allowed to sit for three weeks prior to experiments.

### 16S rRNA gene amplicon sequencing and analysis

The 16S rRNA gene amplicon analysis was performed in collaboration with Prof. T. Clavel (University Aachen) and Prof. K. Neuhaus (University Munich). DNA was extracted from feces using QIAamp DNA stool mini kits and 24 ng of genomic DNA was used for amplification (25 cycles) of the V3/V4 region of the 16S rRNA genes using the bacteria-specific primers 341 F and 785 R.^63^ Purification of amplicons was performed using the AMPure XP system (Beckmann) and sequencing was performed in paired-end modus (PE275) with pooled samples in a MiSeq system (Illumina Inc.) according to manufacturer’s instructions and a final concentration of 10 pM DNA and 25% [v/v] PhiX standard library. Demultiplexing and OTU (operational taxonomic unit) clustering from raw 16S rRNA gene amplicon dataset was performed with IMNGS.^64^ The similarity cutoff for OTU clustering in imngs was set to 97% identity. Parameters for the imngs analysis were set as: two mismatches are allowed in the barcode, minimum fastq quality score of three for trimming of unpaired reads, 350 to 550 base pairs length for amplicons for paired overlapping sequences, maximum of four expected errors in paired sequences, ten base pairs length of trimming at the forward and reverse side of the sequences, 0.5 % relative abundance of OTU cutoff. Downstream analysis for abundance and taxonomic classification of OTUs contains was performed with the R script set Rhea.^65^

### Quantitative PCR for Oligo-MM12 consortium

Genomic bacterial DNA of fecal material was isolated using the QIAamp DNA Stool mini Kit (Qiagen) following the manufacturer’s instructions. For quantification of the Oligo-MM^12^ consortium and *A. finegoldii* 17242 within the intestinal microbiota we used the hydrolysis-probe-based qPCR published by Brugiroux and colleagues.^22^ Duplex qPCRs were run using the LightCycler® 480 Probes Master (Roche) and LightCycler® 480 Instrument II (Roche). For standard curve preparation the concentration of sequence copy numbers was calculated on plasmids containing 16S rRNA genes. Ten-fold serial dilutions (10^8^–10^−2^ copies/µl) were prepared in water supplemented with 100 ng/µl yeast t-RNA and used for absolute quantification of the copy number.

### Intestinal transit time

The whole gut transit time was measured in accordance with the protocol of Nagakura and colleagues.^66^ One part of each diet was mixed with five parts of sterile water and 6% [w/v] carmine as a maker. Mice were fasted for 6 hours and fed with 300 µl of the marked diet. The time between oral administration and the first red-colored fecal pellet was measured.

### Experimental colitis model

Dextran sulfate sodium (DSS, MP Biomedicals) was used to induce an acute colitis. Mice were exposed to DSS in the drinking water for five days, as indicated in the figure legends. Analysis were performed on day five or six following colitis induction. Control mice received conventional drinking water.

### Colon ex vivo explant culture

Approximately 1 cm of colonic tissue was washed in 1 ml RPMI cm (100 rpm, 37°C, 20 min). Afterward the gut was opened longitudinally and cultured in 20 µl/mg RPMI-cm at 37°C and 5% CO2 for 24 hours. Supernatants were harvested, centrifuged and stored at 20°C.

### ELISA

The Lipocalin-2/NGAL DuoSet ELISA, mouse (R&D Systems), IL-18 ELISA Kit (Sino Biological) and TNF alpha Mouse ELISA Kit (invitrogen) were performed according to manufacturer’s instruction.

### Bead-based multiplex immunoassays

Simultaneous quantification of multiple cytokines was performed with the BioLegend Legendplex™. The mouse Th17 Cytokine Panel (8-plex) Legendplex™ assay was performed according to manufacturer’s instruction. Samples were measured at the Attune NxT Flow Cytometer. The data were analyzed using the LEGENDplex™ Data Analysis Software.

### Histology and fluorescence in situ hybridization (FISH)

The water-free methacarn (methanol-Carnoy’s) fixation was used to preserve the intestinal mucus layer during histological preparation.^67^ Intestinal tissues with fecal pellets were fixed over night with methacarn at room temperature. Tissues were washed in methanol for 30 min, two times in ethanol for 15 min, once in ethanol/xylene (1:1) for 15 min and two times in xylene for 15 min prior to embedding in paraffin.

For periodic-acid-Schiff (PAS) histology, 3–5 µm thin tissue sections were dewaxed and stained for 10 min with periodic acid solution (0.5%, w/v; Merck), 20–30 min in Schiff’s reagent (Merck) and 1 min in Mayer’s Hämalaun (Carl Roth). Between each step, sections were rinsed in running water.

For FISH analysis, 3–5 µm thin tissue sections were dewaxed and then treated with 50 µl 4% lysozyme solution (45 min, 37°C) to demask nucleic acids. After washing, 50 µl hybridization solution was added and incubated for 3 hours at 50°C. Slides were washed several times at 37°C and were dried at RT before mounted with ProLong™ Gold Antifade Mountant with DAPI (Thermo Fisher Scientific) following manufacturer’s instructions. The samples were documented at a Leica DM 5500 wide field microscope (Leica) and analyzed with ImageJ (National Institutes of Health).

### Cell isolation techniques

Single cell suspensions were performed from spleen and mLN by mechanical disruption and passage through filter (Milteny Biotec). Lamina propria mononuclear cells (LPMCs) of cells were isolated using the Lamina Propria Dissociation Kit (Miltenyi Biotec) according to manufacturer’s instructions. In short, tissues were transferred to preheated digestion solution in C tubes (Miltenyi Biotec) and processed by gentleMACS Octo Dissociator (Miltenyi Biotec). The obtained cell suspension was filtered on a 100 µm cell strainer and washed with PB buffer. Cells were centrifuged (300 x g, 4°C, 10 min) prior to cell counting and following procedures.

### Cell staining procedures and Flow cytometry

To exclude dead cells, Zombi NIR™ viability dye was used (BioLegend) prior incubation with antibodies at recommended dilutions for 20 min at 4°C. For intracellular staining of transcription factors, cells were fixed and permeabilized. After cell surface staining, cells were washed with 1 x PBS and fixed with the Foxp3 Fixation Kit (BioLegend) for 20 min at 4°C. After washing with PBS/1% FCS and saponine buffer, cells were incubated with antibodies diluted in saponine buffer for 40 min at 4°C. After further washing with saponine buffer and PBS/1% FCS cells were analyzed by flow cytometry. For intracellular cytokine staining, cells were stimulated for 4 h with PMA (50 ng/ml) and ionomycin (750 ng/ml) in the presence of Brefeldin A (5 µg/ml, Sigma). After cell surface staining, cells were washed with PBS and fixed in 2% formaldehyde for 30 min at RT. Following washing with PBS/1% FCS and saponine buffer, cells were incubated with antibodies diluted in 100 µl saponine buffer for 20 min at 4°C. Flow cytometric analysis was performed after further washing steps, using Attune NxT Flow Cytometer (Thermofisher).

### HPLC-MS quantification of cellotetraose degradation products

Digestion of water soluble cellotetraose was performed with supernatants of cecal content containing enzymes. To obtain enzymes, fecal content was dissolved in PBS containing 1% [v/v] 100 x HALT™ Protease Inhibitor Cocktail (Thermo Fisher Scientific) (300 µl/100 mg) and incubated on ice for 10 min. The luminal material was homogenized and centrifuged twice (3,500 x g, 4°C, 20 min). The clear supernatant containing cellulolytic enzymes was 1.5-fold diluted and used for enzymatic assays. The protein solution was mixed with cellotetraose resulting in a final concentration of 200 µM and incubated for 30 and 60 min at 37°C. As controls, cecal supernatants were heat inactivated at 95°C for 15 min prior to addition of cellotetraose. The reaction was terminated by freezing in liquid nitrogen. Samples were stored at −20°C before HPLC analysis.

After thawing, the samples were heat inactivated (95°C, 15 min) to exclude further enzymatic processes. The samples were centrifuged (3,500 x g, 4°C, 20 min) and 10-fold diluted. 50 µl of this solution was transferred to a HPLC-column EC 125/2 NUCLEODUR (Macherey-Nagel) and the column temperature was set to 25°C. The elution was performed using an Agilent 1100 HPLC system with the following gradient of water and acetonitrile: isocratic elution with 2% acetonitrile for 5 min, followed by a linear increase to 10% acetonitrile within 5 min and to 85% in additional 10 min. During the next 10 min, the amount of acetonitrile was increase linearly up to 95% and hold for 5 min more. The HPLC system was directly connected to an LTQ-FT-Ultra mass spectrometer (Thermo Fisher Scientific) equipped with an electrospray ion source. Mass spectrometric detection was carried out in positive ion mode.

After thawing, the samples were heat inactivated (95°C, 15 min). The samples were centrifuged (20 x g, 4°C, 20 min) and 25 µL of the supernatant was transferred to sample vials. CE-MS analysis was performed using an Agilent 1600A Capillary Electrophoresis System equipped with a 100 cm fused silica capillary (ID 50 µM) coupled to an Agilent 6120 single quadrupole mass spectrometer. The method was previously described by Klampfl et al.^68^ As background electrolyte 300 mM triethlyamine was used and 80% isopropanol supplemented with 0.25% triethylamine was provided at a flow rate of 4 µL/min as sheath liquid. The sample was injected by pressure (50 mbar, 9 s) and separated by the application of 20 kV separation voltage. Detection of the analytes was performed in negative ion mode with capillary voltage set to −5000 V. During the analysis, the nebulizer was switched off and the drying gas flow was set to 1.5 L/min at 150°C.

### UHPLC-MS quantification of cecal short-chain fatty acids and bile acids

The analysis of bile acids and short chain fatty acids was performed as published previously.^69,70^ Cecal content was isolated, frozen in liquid nitrogen and stored at −80°C. To avoid bacterial metabolism, further steps were performed on dry ice, including cutting for weighting. Cold Methanol was added prior homogenization and centrifugation. The Acquity UHPLC system (Waters) and a UHPLC column (Acquity™ UPLC BEH™ C8, Waters) were used for UHPLC. Mass spectrometry was performed with the amaZon ETD Ion Trap (Bruker Daltonics GmbH) in negative ionization mod and the maXis (Bruker Daltonics GmbH) in positive electorspray ionization mode for SCFAs.

### Quantitative PCR for murine transcripts

Colonic tissue (1 cm) was added to 1.0 ml Extrazol (Biolab Innovative Research Technologies) and stored at −80°C or used directly for homogenization with the Ultra-Turrax (IKA) homogenizer. The total RNA isolation was performed according to manufacturer’s instructions. Afterward, the RNA was treated with the TURBO DNA-free™ Kit (invitrogen, Thermo Fisher Scientific) following the manufacturer’s routine DNase treatment protocol. The RNA concentration was measured using NanoDrop system (Thermo Fisher Scientific). cDNA was synthesized from 500 ng RNA using the RevertAid First Strand cDNA Synthesis Kit (Thermo Fisher Scientific) following the manufacturer’s instructions. Reaction mix without RevertAid M-MuLV RT was used as a control for genomic DNA contamination. RT-PCRs were run using the qPCR Core kit for SYBR® Green I (Eurogentec) according to manufacturer’s instructions. Samples were run in duplicates. The specificity of the amplicon was confirmed by melting curve analysis. To control for contamination, the amplification of controls for genomic DNA were evaluated and non-template control (PCR-grade water) was included in every run. Via ΔΔCt method the relative quantity of target DNA was quantified using glycerinaldehyd-3-phosphat-dehydrogenase (GapDH) as housekeeping gene.

### Transcriptional profiling

Epithelial cells were isolated from the colon by vigorous shaking after incubation in DMEM/10% FCS/1 mM DTT and two times in HBSS/5 mM EDTA. All cell fractions were pooled and separated from debris by a 20/40%-percoll gradient. Cells were resuspended in RLT-Buffer containing b-mercaptoethanol. The RNA was isolated and sequenced as published previously (37). In short, RNA was purified using the RNeasy Plus Micro Kit according to the manufacturer’s instructions (Qiagen). Libraries were prepared from samples with an RNA integrity number (RIN) greater than 8 and barcoded using NEBNext Poly(A) mRNA Magnetic Isolation Module and NEBNext Ultra II RNA Library Prep Kit for Illumina (New England Biolabs) according to the manual. Barcoded RNA-Seq libraries were onboard clustered using HiSeq Rapid SR Cluster Kit v2 using 8 pM and 59 bps were sequenced on the Illumina HiSeq2500 using HiSeq Rapid SBS Kit v2 (59 cycle). The raw output data were preprocessed according to the Illumina standard protocol. After initial quality assessment, raw output fastq sample files were trimmed and mapped with the Qiagen CLC Workbench v. 10.0.1, using the murine genome version GRCm38. Total read counts were further processed in R using the DeSeq2 package to compute differential gene expression and adjusted *P* values. Unsupervised clustering was performed based on gene sets published by Haber et al. (15).

### Statistics

The data were analyzed with GraphPad Prism 8. Significance was calculated using tests indicated in the figure legends. Values less than 0.05 were considered statistically significant.

## Supplementary Material

Supplemental Material
