# Supplementary material for: Dietary cellulose induces anti-inflammatory immunity and transcriptional programs via maturation of the intestinal microbiota
Source: Gut Microbes. 2020 Oct 20;12(1):1829962. doi: 10.1080/19490976.2020.1829962 (PMC7583510; doi:10.1080/19490976.2020.1829962)
Supplement: Supplemental Material [file KGMI_A_1829962_SM8476.zip › Supplementary information/Cellulose_supplements.docx]

# Supplementary Figures

Figure S1. Impact of diet on growth and development of mice. **(a)** Composition of purified diets containing 7% cellulose as only dietary fiber (CD) or free of any fiber (FFD). **(b)** Weight gain of CD and FFD mice (n = 5). **(c)** Intake of the diet shown as gram per mouse and day (n = 6). (d) Intestinal transit time after fasting (n = 4). Statistical analysis: (b) two-way ANOVA and (c, d) unpaired, two-tailed Student’s t test. Data are shown as individual mice and means ± SD.

Figure S2. The impact of cellulose on the diversification of the intestinal microbiota. The diversity of the intestinal microbiota of twenty-five-week-old B6 mice was analysed by 16S rRNA gene amplicon analysis (n = 3). (a) Diversity shown as multi-dimensional scaling (MDS) plot based on generalized UniFrac dissimilarities. (b) Alpha diversity and (c) the Firmicutes/Bacteroidetes ratio. Statistical analysis: (a) non-parametric multivariate analysis of variance (Rhea), (b, c) unpaired, two-tailed Student’s t test. Data are shown as individual mice and means and are representative of two independent experiments.

Figure S3. The influence of dietary cellulose on the bacterial metabolome. Caecal samples were isolated from SPF and germ-free B6 mice receiving either CD or FFD from birth (SPF) or four weeks prior to analysis (germ-free). (a) Short chain fatty acids, (b) primary conjugated, (c) unconjugated and (d) secondary bile acids were measured via UHPLC‑MS (n = 4‑6). Statistical analysis: (a-d) unpaired, two-tailed Student’s t test (corrected for multiple comparison by Sidak-Holm method). Data are shown as individual mice and means and are representative of two independent experiments. *p < 0.03, **p < 0.002, ***p < 0.0002 and ****p < 0.0001.

Figure S4. Influence of FFD on degradation of cellotetraose. Signals intensities (AUC) of degradation products after incubation of cellotetraose with native and heat-inactivated cecal enzymes in FFD a (n = 1). Data are shown as means + SD and are representative of two independent experiments.

Figure S5. Lymphocytes from spleen, mLN and intestinal lamina propria of CD and FFD mice were analysed by FACS. (a) Numbers of total lymphocytes and (b) frequencies of total CD4+ T cells. (c) Faecal sIgA of CD and FFD mice. Statistical analysis: (a, b) unpaired, two-tailed Welch’s test and (c) unpaired, two-tailed Student’s t test. Data are shown as individual mice and means + SD and are representative of two independent experiments.

Figure S6. Influence of dietary cellulose on development of colitis on transcriptional profiles of gut epithelial cells. B6 mice received CD or FFD from birth and were treated with 1.5 % or 2.5 % DSS for five days. (a) The diarrhea score was quantified as follows: 0, normal stool; 1, soft stool or traces of blood; 2, very soft stool with traces of blood; 3, watery stool, rectal bleeding (n = 3-4). (b) Macroscopic appearance of the colon (2.5% DSS). (c) TNFα and lipocalin-2 were measured in ex vivo in colon cultures of 1.5% DSS-treated mice via ELISA (n = 3‑6). RAG KO mice received CD or FFD for four weeks and following treatment with 1.5 % DSS for five days. (d) Unsupervised clustering of genes related to distinct intestinal epithelial subtypes differentially expressed in CD and FFD (RAG KO) mice (n = 5-6). (e) Weight loss and (f) colon length/weight ratio (n = 5‑6). Statistical analysis: (a, f) unpaired, two-tailed Student’s t test, (c) unpaired, two-tailed Mann-Whitney test and (e) two-way ANOVA. *p < 0.03, **p < 0.002, ***p < 0.0002, and ****p < 0.0001. Data are shown as individual mice and (a, e, f) means + SD or (c) median + CI and are representative of two independent experiments.

Figure S7. Impact of A. finegoldii on intestinal immune system and colitis. Lymphocytes of A. finegoldii colonized Oligo-MM12 mice were isolated from peripheral lymphoid organs and intestinal lamina propria. (a) The number of lymphocytes isolated from the ileal and colonic lamina propria. (b) The frequency of CD4+ T cells in indicated organs. CD4+ T cells were analyzed for (c, d) cytokines IL-17 and IFN‑γ as well as (e, f) transcription factors RORγt and FOXP3 (n=4-6) by FACS. Cytokines from colon cultures were measured by LEGENDplexTM and ELISA (g) under homeostasis and (h) after treatment with 3.5 % DSS for five days. Statistical analysis: (a, b, c, e) unpaired, two-tailed Welch’s test and (g, h) unpaired, two-tailed Mann-Whitney Test. Data are shown as individual mice and (a, b, d, e) means + SD or (g, h) median + CI and are representative of two independent experiments.
