# Supplementary figures and images for: Dietary cellulose induces anti-inflammatory immunity and transcriptional programs via maturation of the intestinal microbiota
Source: Gut Microbes. 2020 Oct 20;12(1):1829962. doi: 10.1080/19490976.2020.1829962 (PMC7583510; doi:10.1080/19490976.2020.1829962)

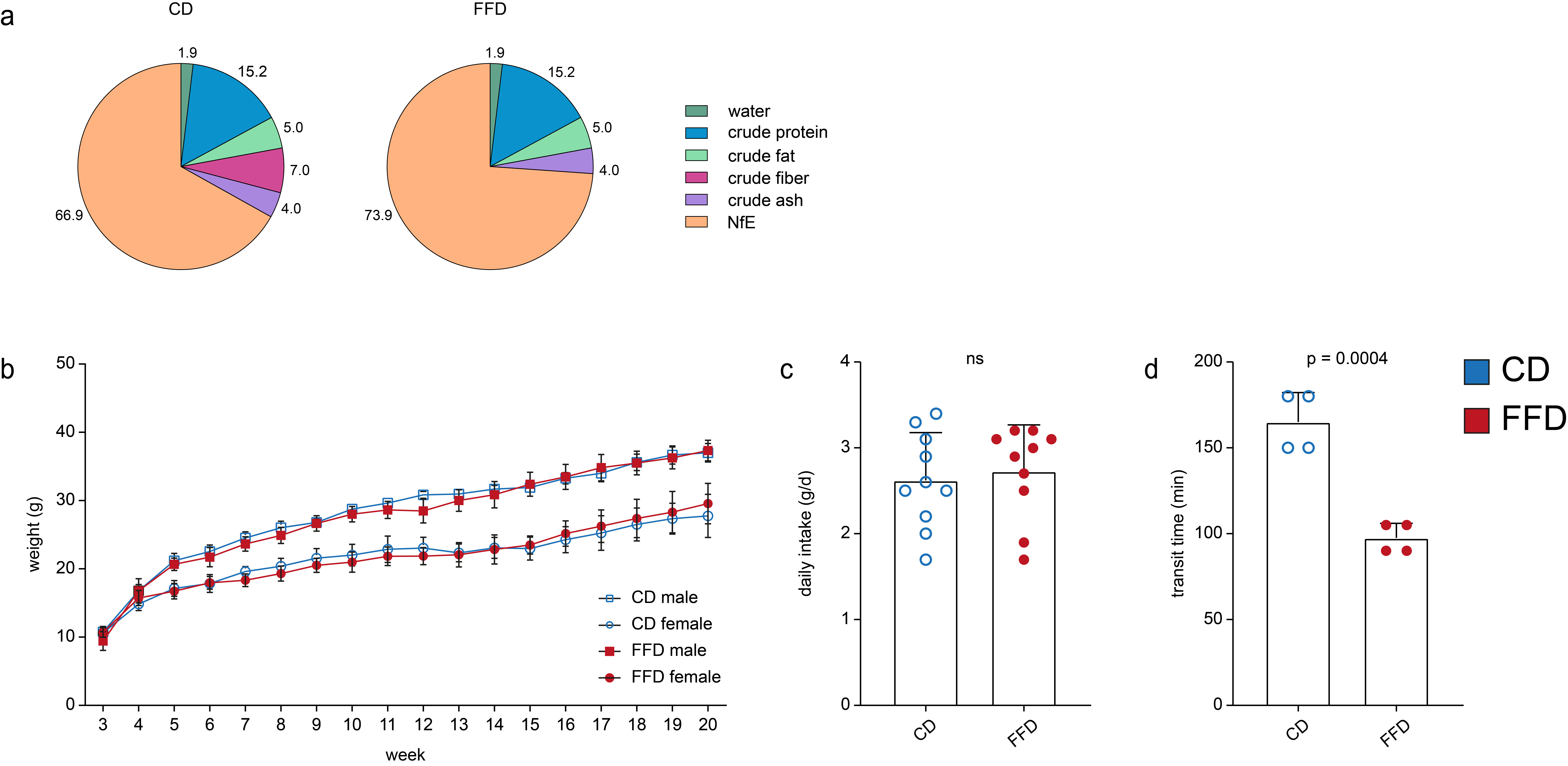

Supplement: Supplemental Material [file KGMI_A_1829962_SM8476.zip › Supplementary information/Figure S1.jpg]

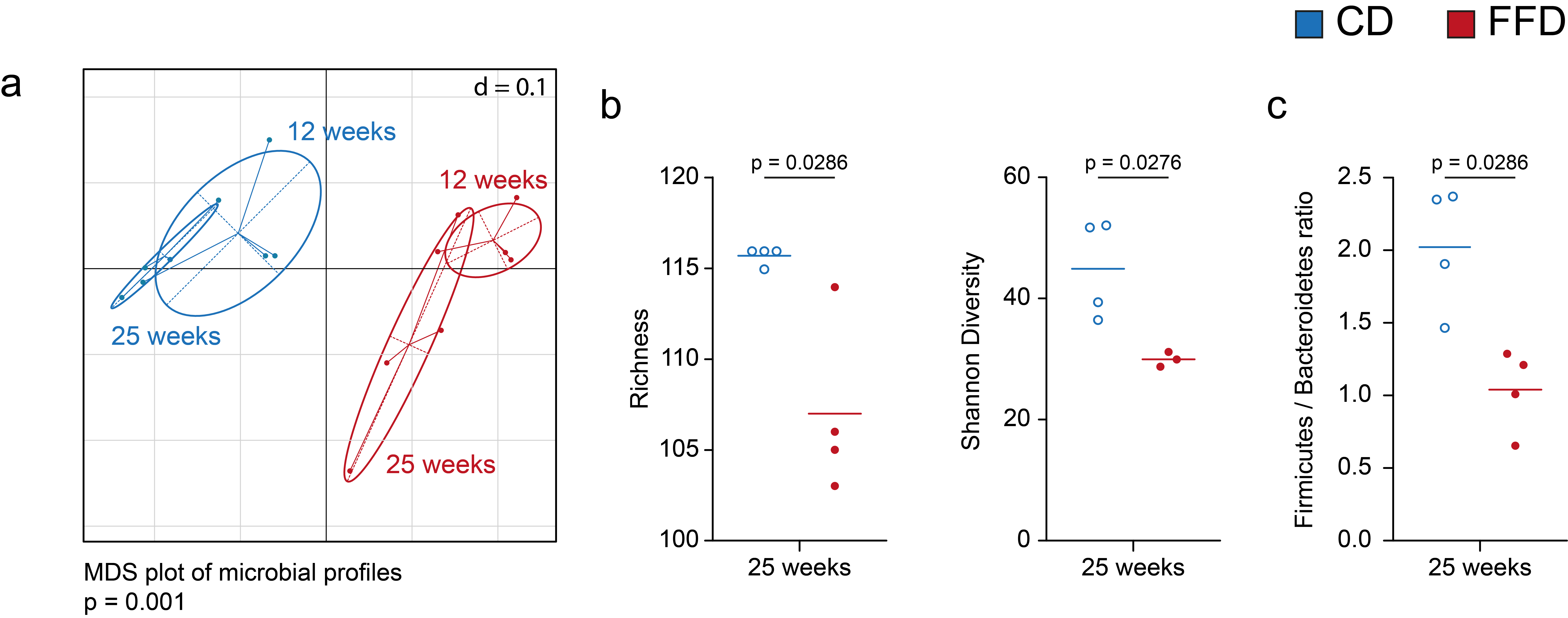

Supplement: Supplemental Material [file KGMI_A_1829962_SM8476.zip › Supplementary information/Figure S2.jpg]

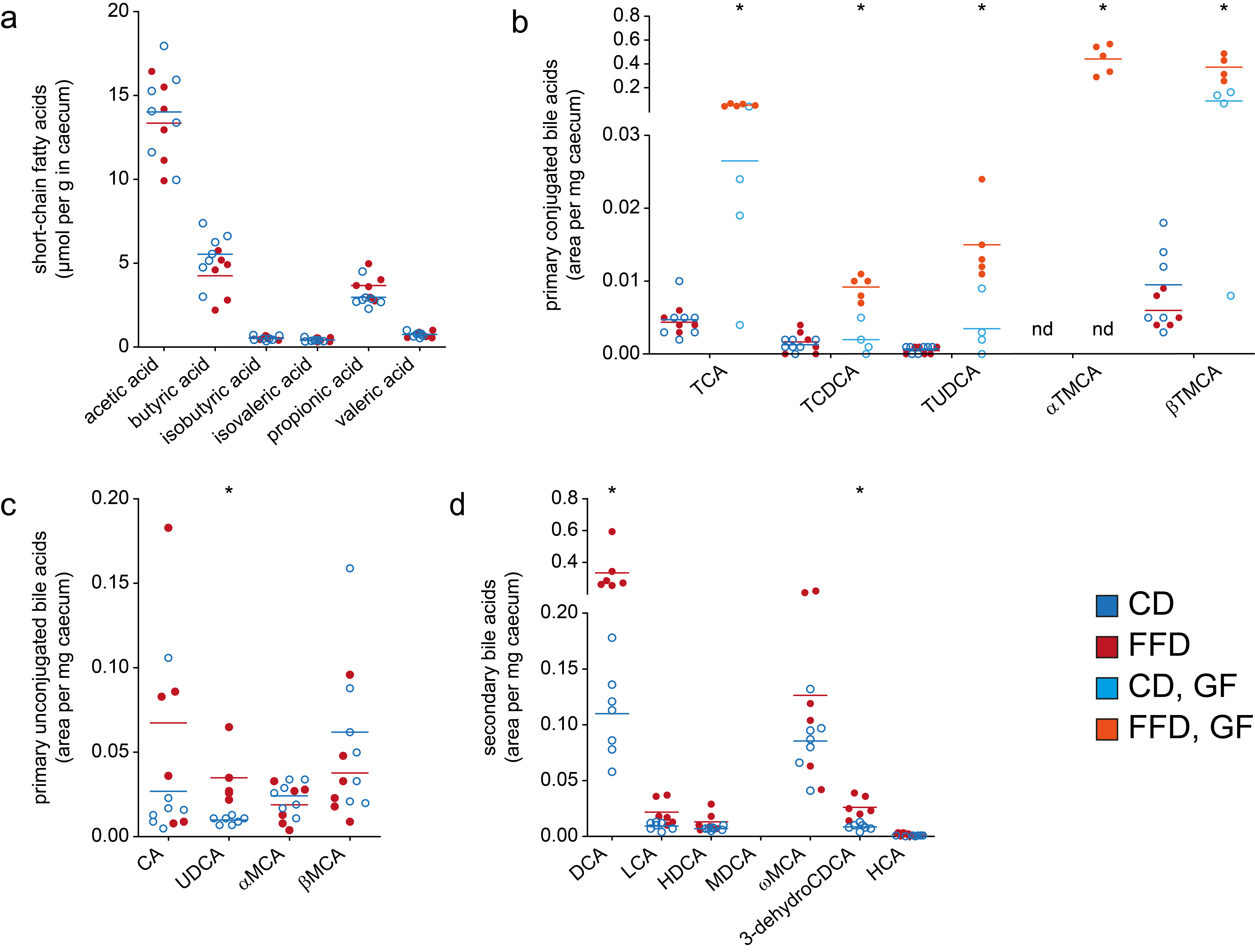

Supplement: Supplemental Material [file KGMI_A_1829962_SM8476.zip › Supplementary information/Figure S3.jpg]

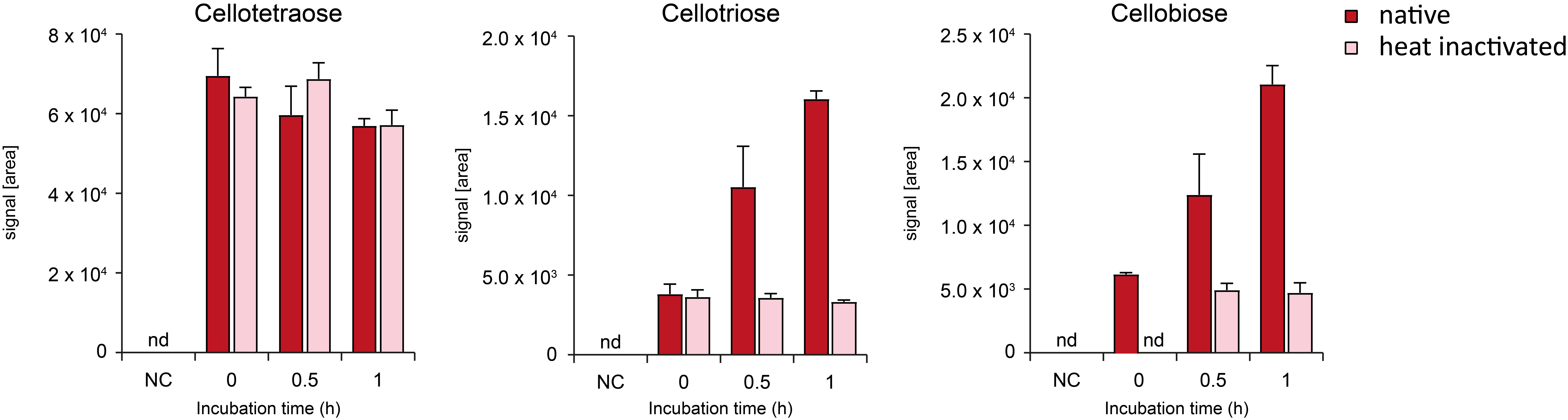

Supplement: Supplemental Material [file KGMI_A_1829962_SM8476.zip › Supplementary information/Figure S4.jpg]

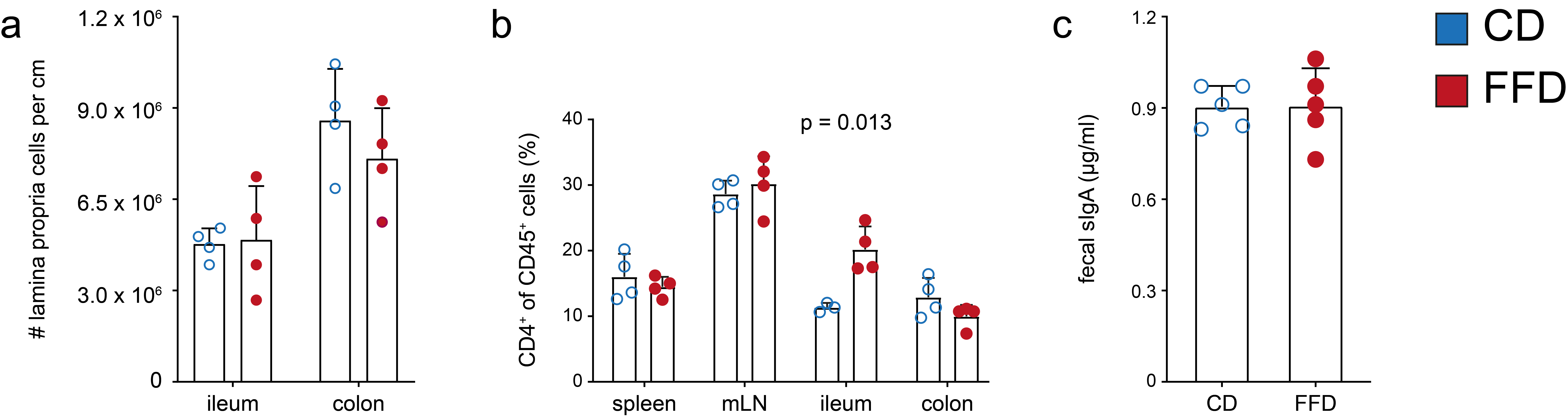

Supplement: Supplemental Material [file KGMI_A_1829962_SM8476.zip › Supplementary information/Figure S5.jpg]
